# Identification of the Sex Pheromone of the Asparagus Moth, *Parahypopta Caestrum* (Lepidoptera, Cossidae)

**DOI:** 10.1007/s10886-024-01504-y

**Published:** 2024-05-20

**Authors:** Onofrio Marco Pistillo, Ilaria D’Isita, Antonella Di Palma, Giacinto Salvatore Germinara

**Affiliations:** Department of Agricultural Sciences & Food, Natural Resources and Engineering, University of Foggia, Foggia, Italy

**Keywords:** Cossidae, Sex pheromone, GC-MS-EAD, (*Z*)-5-tetradecenyl acetate, (*Z*)-7-tetradecenyl acetate, (*Z*)-9-tetradecenyl acetate, Field trapping

## Abstract

Chemical, electrophysiological, and field trapping experiments were carried out to identify the female-produced sex pheromone of the asparagus moth, *Parahypopta caestrum*, a very serious pests of asparagus cultivations in southern Europe. Gas chromatography coupled with mass spectrometry and electroantennogram detection (GC-MS-EAD) analysis of hexane and solid-phase microextraction (SPME) extracts of sex pheromone glands of calling females consistently detected four compounds eliciting EAG responses in male moth antennae. According to their GC retention times, mass spectra, and comparative EAG analyses with reference standards, these EAD-active compounds were identified as (*Z*)-9-tetradecenol (*Z*9-14:OH), (*Z*)-5-tetradecenyl acetate (*Z*5-14:Ac), (*Z*)-7-tetradecenyl acetate (*Z*7-14:Ac), and (*Z*)-9-tetradecenyl acetate (*Z*9-14:Ac), respectively. In the SPME extracts from the head-space of individual abdominal tips, *Z*9-14:Ac, *Z*5-14:Ac, *Z*7-14:Ac, and *Z*9:14 OH were detected in the ratio of 82:9:5:4. In EAG dose-response experiments, *Z*9-14:Ac was the strongest antennal stimulant at different doses tested. In field trapping experiments, *Z*9-14:Ac, *Z*7-14:Ac, and *Z*5-14:Ac proven to be essential for male attraction and a their 85:5:10 blend loaded onto green rubber septum dispensers was significantly more effective than single-, two-, and any other three-component blend of these compounds. The addition of *Z*9-14:OH to the optimal blend resulted in a significant reduction of male catches. The attractive blend here identified allowed for an effective and accurate monitoring of *P. caestrum* flight activity in southern Italy.

## Introduction

*Parahypopta caestrum* (Hübner) (Lepidoptera, Cossidae) is a monovoltine species of Eurasian origin spread in coastal and hilly areas of southern Europe specifically infesting *Asparagus* spp. including *A. officinalis, A. acutifolius, A. albus, A. maritimus* (Tarasco 2002). Eggs are laid in groups in the soil and larvae bore roots and stems causing very serious damages particularly to young plants that results in crop thinning during the first 2–3 years after transplanting (Pollini 1989). In the Mediterranean basin, the asparagus moth is recognised as one of the main limiting factor of asparagus crops (Tarasco 2002; Salpiggidis et al. 2004). For example, in Foggia province (southern Italy), the widest area of green asparagus production in Italy, *P. caestrum* highly infests approximately 10,000 ha and the severity of its damage is threatening the continuation of asparagus cultivation in this area (Tarasco et al. 2002). Control of this pest is difficult due to its cryptic nature, the endophytic development of larvae, the lack of in-depth knowledge on pest biology and the unavailability of effective chemical control means. Therefore, the development of effective monitoring and low-impact control tools are urgently needed.

The identification of the female-produced sex pheromone could provide a useful tool to monitor the flight activity of *P. caestrum* adults and develop semiochemical-based control means. Among Cossidae, sex-pheromones have been identified for many related species including *Zeuzera pyrina* (L.) (Tonini et al. 1986), *Cossus cossus* (L.) (Capizzi et al. 1993); *Holcocerus insularis* (Zhang et al. 2001), *Holcocerus hippophaecolus* Hua (Fang et al. 2005), *Cossus insularis* (Staudinger) (Chen et al. 2006), *Holcocerus arenicola* (Staudinger) (Jing et al. 2010), *Holcocerus artemisiae* Chou et Hua (Zhang et al. 2009), *Isoceras sibirica* (Alpheraky) (Zhang et al. 2011), *Holcocerus vicarious* (Walker) (Yang et al. 2015), and *Coryphodema tristis* (Drury) (Bouwer et al. 2015). The common structures of sex pheromones identified in Cossidae are monounsaturated docenyl acetates or tetradecenyl acetates with the double-bond in position 3, 5, 7 or 9 or diunsaturated tetradecadienyl acetates with the conjugated double-bond in position 3, 5 (El-Sayed 2023). Nonetheless, sex pheromone of *P. caestrum* has not been characterized. To this aim, in the present study different extracts from pheromone gland of calling females were analysed by gas-chromatography coupled with mass-spectrometry and electroantennogram detection (GC-MS-EAD). The EAG technique using authentic standards was employed to confirm configuration of the EAG-active compounds and to study the male antennal sensitivity to these compounds. Finally, field trapping experiments with individual synthetic compounds and their different blends were carried out to define an effective trap lure.

## Materials and methods

### Insects

Cocoons of *P. caestrum* were collected in highly infested asparagus crops in Foggia province (Apulia region, Italy) during May 2020–2022. Pupae were individually kept in transparent plastic cups (Ø 6 cm × 8 cm) and maintained at 23 ± 2 °C, 60 ± 5% r.h., and L14:D10 photoperiod in a climatic chamber. Newly emerged adults were transferred in clean transparent plastic cups covered with a fine mesh net (1 mm) and provided with a piece of cotton soaked with a 10% sucrose solution.

### Female Calling

The observation of the diel rhythm of calling behaviour was carried out on 1- to 3-day-old virgin females (*n* = 20) held under the environmental conditions described above. Female calling was quantified by counting the number of females assuming the typical posture of abdomen protrusion at 1-h intervals during the scotophase.

### Solvent Extraction

The terminal abdominal segments including the pheromone gland were excised from virgin females that had been calling for 1 h during the second scotophase. Individual abdominal tips were immersed in a 0.5 mL glass conical vial (Supelco, Inc. Bellefonte, USA) containing 100 µL of *n*-hexane (Sigma Aldrich, Milan, Italy) for 30 min at room temperature. Extracts from 5 abdominal tips were transferred to a clean conical vial and concentrated to 1 female equivalent per µL (FE µL^–1^) using a gentle stream of nitrogen. For pheromone titer determination, additional extracts (*n* = 10) were prepared from individual abdominal tips. Extracts were stored at − 20 °C until needed.

### Solid Phase Micro-extraction (SPME)

An abdominal tip of a calling female was dissected after tying it with a cotton thread at the fifth urites to reduce the impurities arising from the haemolymph (Rotundo et al. 2001). The tip was placed inside a conical vial (0.5 mL) previously silanised using trimethylchlorosilane (TMCS) and closed with a PTFE/silicone septum screw top cap (Agilent Technologies). A 50/30 µm divinylbenzene–carboxen–polydimethylsiloxane (DVB–CAR–PDMS) SPME fiber (Supelco Co., Bellefonte, PA, USA), previously conditioned in a gas-chromatograph (GC) injection port at 270 °C for 1 h, was exposed to the headspace of the sample for 30 min at room temperature. Then, the SPME needle was retracted in the holder and desorbed in the injector of GC. Five replicates of the SPME extract were prepared.

### Chemicals

Series of monounsaturated *cis* (*Z*) and *trans* (*E*) aliphatic C_14_ acetates and alcohols (purity > 98%) used for electrophysiological experiments were purchase from the Pherobank (Wageningen, The Netherlands). (*Z*)-7-dodecenyl acetate (*Z*7-12:Ac) (purity ≥ 98%) was purchased from Novapher (San Donato Milanese, Milan, Italy) and used as an external standard to obtain a calibration curve. For field trials, (*Z*)-9-tetradecenol (*Z*9-14:OH), (*Z*)-7-tetradecenyl acetate (*Z*7-14:Ac), (*Z*)-5-tetradecenyl acetate (*Z*5-14:Ac), and (*Z*)-9-tetradecenyl acetate (*Z*9-14:Ac) (purity ≥ 98%) were supplied by Chemface (Wuhan, Hubei, China).

### Gas Chromatography Coupled with Mass Spectrometry and Electroantennogram Detection (GC-MS-EAD)

Solvent and SPME extracts were analysed by GC-MS-EAD using a 7890B GC equipped with a split/splitless injector and an HP-5MS capillary column (30 cm × 0.25 mm i.d., × 0.5 μm film thickness, J&W Scientific Inc., Folsom, CA, USA), linked to a 5977 A quadrupole mass detector (Agilent technologies, Palo Alto, USA). GC conditions were: carrier gas helium at 1.25 mL/min; injector temperature, 250 °C; splitless time, 30 s for solvent extracts and 4 min for SPME extracts; oven program, from 60 to 250 °C at 5 °C/min, 250 °C for 15 min. The effluent from the column was equally splitted by a Graphpack 3D/2 flow splitter (Gerstel, Mülheim, Germany) between the MS and a 30 cm long transfer line (Effluent Conditioner Assembly, Type EC-03, Syntech Laboratories, Hilversum, The Netherlands) by an 83.9 cm × 0.1 mm i.d. and a 54.7 cm × 0.15 mm i.d. deactivated capillary, respectively. Length and internal diameter of deactivated capillaries were determined by using the Gerstel ODP column calculator software (Gerstel, Germany). The temperature of the transfer line was regulated by a digital temperature control (Type TC-02, Syntech Laboratories, Hilversum, The Netherlands) set at 250 °C. At the end of the transfer line, the column effluent was mixed to a constant flow of charcoal-filtered humidified air (500 mL/min) passing in a glass tube (i.d. 6 mm x 10 cm) with the outlet positioned about 1 cm from a male moth antennal preparation (see below). The analog EAD signal was amplified (10x) and converted into a digital signal using the converter ProbeAmp (Syntech Laboratories, Hilversum, The Netherlands) and recorded at 10 Hz/0.02 min rate with a data channel of the GC-MS using the GC ChemStation software in addition to the MS ChemStation software (Agilent technologies, Palo Alto, USA). Mass spectra were recorded in the electron impact mode (ionization energy, 70 eV) in a range of 15–550 amu at 2.9 scans/s. For solvent extracts, a solvent delay time of 4 min was used. Solvent controls were analyzed to check for interferences. EAD-active compounds were identified by observing characteristic ions and comparing their mass spectra with those of the data system library NIST11 (*p* > 90%) and by comparing their retention times and mass spectra with those of authentic standards. For quantification of EAD-active compounds, an external standard calibration curve was calculated using concentrations of 1, 5, 10, 30, and 50 ng/µL of (*Z*)-7-dodecenyl acetate (R^2^ = 0.99). The quantification was carried out in Selected Ion Monitoring mode (SIM) using the m/z 81 ion to maximize the sensitivity for detection of any trace compound. Then, solvent extracts from individual abdominal tips were analysed in SIM mode (m/z 81 ion) and the EAD-active peaks quantified by comparing their integrated peak areas with those of the external standard curve at comparable relative responses. Concentration of each EAD-active compound is expressed as ng of (*Z*)-7-dodecenyl acetate equivalents per female.

### Electroantennography (EAG)

To contribute to chemical characterization of putative sex-pheromone components and to evaluate the male antennal sensitivity to these compounds the EAG technique was used (Rotundo et al. 2004; Germinara et al. 2007; Germinara et al. 2017, 2019). An antenna was excised at the base from a 1-day-old male and, after removing the two distal segments, it was mounted between two glass electrodes filled with Kaissling’s saline (Kaissling and Thorson 1980). Silver wires were used to maintain the electrical continuity between the antennal preparation and an AC/DC high input probe (gain 10x) in DC mode connected to an IDAC-4 (Intelligent Data Acquisition Controller) amplifier and a PC equipped with the EAG Pro program (Syntech Laboratories, Hilversum, The Netherlands). Aliquots (10 µL) of test stimuli were applied to filter paper strips (1 cm^2^, Whatman No.1) placed in Pasteur pipettes (15 cm long). Stimuli were puffed into a constant charcoal-filtered humidified air stream (500 mL/min) flowing through a stainless-steel tube (i.d. 8 mm) with the outlet positioned about 1 cm from the antenna. During 0.1 s, 2.5 mL of vapor from an odor cartridge were added using a disposable syringe. Intervals between stimuli were 1 min. In a first set of experiments, stimuli were 10 µL of 1 µg/µL solutions of monounsaturated C_14_ aliphatic acetates and alcohols, to confirm double-bond position and configuration of sex-pheromone components candidates (Roelofs et al. 1971). In a second set of experiments, stimuli were 10 µL of decimal hexane solutions from 0.0001 to10 µg/µL of the EAG-active compounds identified in pheromone gland extracts, to evaluate the male antennal sensitivity to these compounds. In the first set of experiments, stimuli were randomly sequences while in the second set they were applied in ascending concentrations. At the beginning of the experiment and every four test stimuli, a control (10 µL of hexane) and reference (10 µL of a 100 ng/µL hexane solution of (*Z*)-3-hexen-1-ol) stimulus was applied. Each test compound was tested on 5 antennae of different insects.

The absolute EAG response (mV) was measured as the maximum amplitude of negative polarity deflection (mV) elicited by a stimulus (Light et al. 1992a). To compensate for solvent and mechanosensory artifacts, the absolute EAG response (mV) to each test stimulus was subtracted by the mean response to the two nearest hexane controls (Raguso and Light 1998). To compensate for the decline of the antennal responsiveness during the experiment, the resultant EAG value was then corrected based on the reduction of the EAG amplitude to the standard stimulus (Den Otter et al. 1991).

### Field Test

Field trapping experiments were carried out in an infested asparagus cultivation in Foggia (Apulia region, Italy) during May and June 2020. Novatrap (Novapher, San Donato Milanese, Milan, Italy) made of semi-transparent green polypropylene (Ø 24 × 32 cm) were suspended on metallic poles 0.5 m above the ground and spaced 30 m apart along the field edges. A green rubber septum (Novapher, San Donato Milanese, Milan, Italy) loaded with 1 mg of individual sex-pheromone component candidates or their binary, ternary or quaternary blends was suspended at the centre of the trap entries by an iron wire. Each putative sex pheromone component was dissolved in hexane at the 5 µg/µL concentration and proper volumes of these solutions were used to load rubber septa dispensers. Unbaited traps were used as controls. Treatments were replicated three times in a randomized block design. At 14-day intervals lures were replaced. Trap catches were collected every 3–4 days and insect species determined by examining male genitalia.

### Data Analysis

The mean male EAG responses to the 10 µg dose of *Z* and *E* isomers of monounsaturated C_14_ aliphatic acetates or alcohols were subjected to Shapiro-Wilk test and to Levene’s test to respectively verify the assumptions of normality and variance homogeneity and then to analysis of variance (ANOVA) followed by the Tukey’s HSD (Honestly Significant Difference) test (*P* = 0.05) for mean comparison. The same procedure was adopted to compare the mean male EAG responses to each dose of different EAG-active compounds. In field trapping experiments, male catches per trap were √x + 0.5 transformed to achieve the assumption of normality (Shapiro-Wilk test) and homogeneity of variance (Levene’s test) and subjected to ANOVA followed by Tukey’s test (*P* < 0.05).

## Results

### Diel Rhythm of Female Calling

**Fig. 1:**
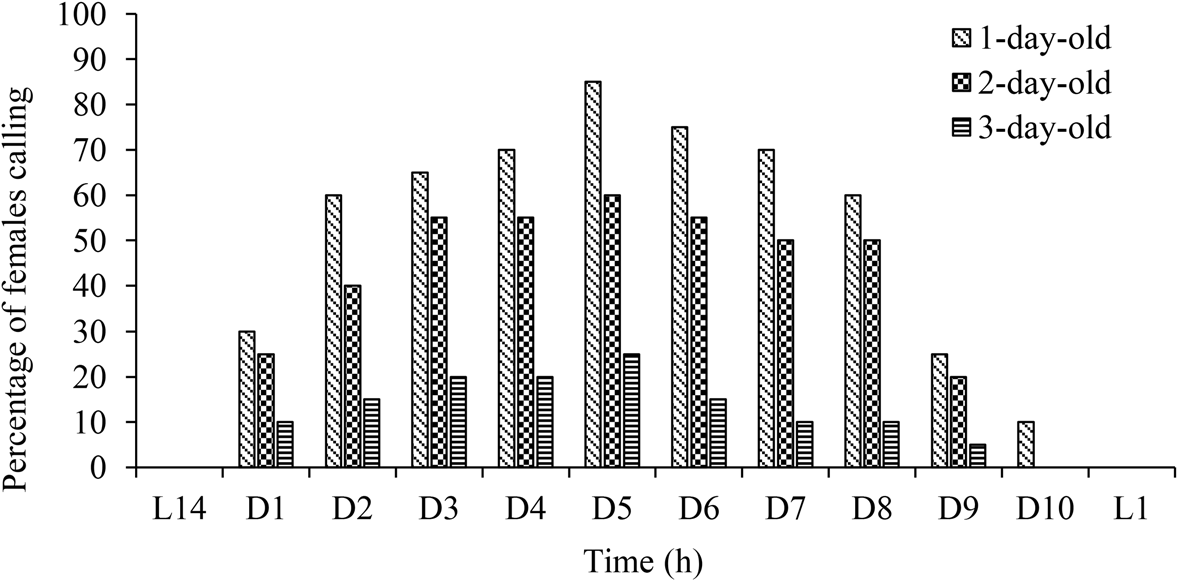
Diel rhythm of calling behavior of *Parahypopta caestrum* virgin females

Females exhibited calling behaviour (extrusion of the last urites) during the first (91%), second (79%) and third (19%) scotophase following emergence. In all scotophases, females started calling during the first hour and calling continued until the beginning of the photophase (D10) (Fig. 1). No calling behaviour was observed before and after scotophase.

### Chemical Analysis

**Fig. 2:**
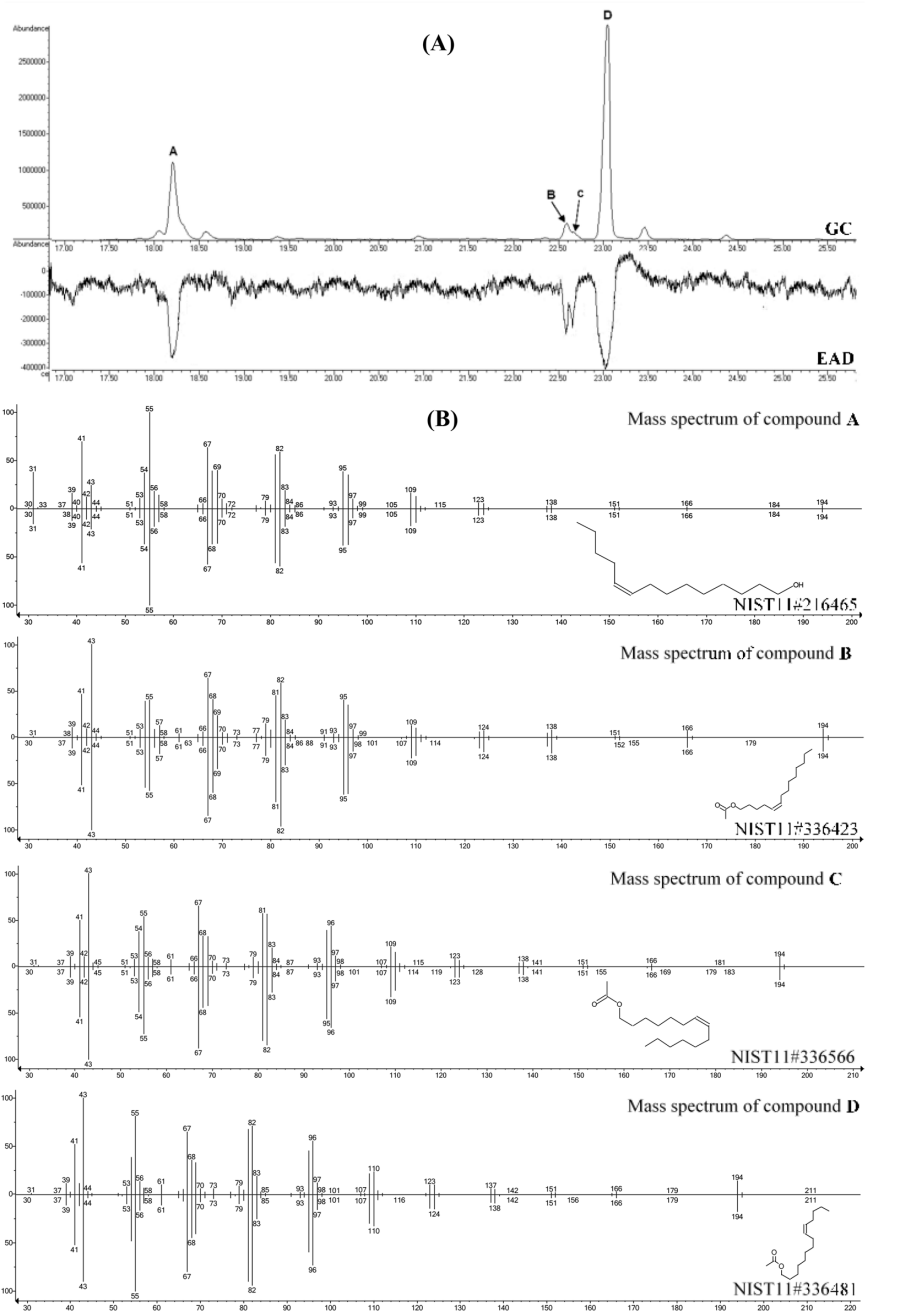
Simultaneously recorded gas chromatographic (GC), mass spectrometric (MS) and electroantennographic detector (EAD male *P. caestrum* antennae) responses to components of pheromone gland extracts. (**A**) Sample GC-MS-EAD response of male antenna to female pheromone gland extracts. In total, four active compounds were found in female pheromone gland extracts and named as compound A, B, C, and D. (**B**) Mass spectra of candidate sex pheromone components in pheromone gland extracts and corresponding best matching in the NIST11 database. Identifications of peaks: (**A**) *Z*9-14:OH; (**B**) *Z*5-14:Ac; (**C**) *Z*7-14:Ac and (**D**) *Z*9-14:Ac

GC-MS-EAD analysis of hexane (1 FE) and SPME extracts revealed four EAD-active peaks at the retention times of 18.15 min (A), 22.51 min (B), 22.63 min (C) and 22.91 min (Fig. 2). The > GC-MS data (Table 1) obtained for the EAD-active components of pheromone gland extracts revealed that the mass spectrum of peak A contained diagnostic ions at *m*/*z* 194 (M^+^−H_2_O), 41 (C_3_H_5_^+^), and 31 (CH_2_OH^+^). This information suggested that compound A was a monounsaturated C_14_ primary alcohol. The mass spectra of peaks B, C, and D showed diagnostic ions at *m*/*z* 194 (M^+^−CH_3_COOH), 61 (CH_3_COOH_2_^+^), 43 (CH_3_CO^+^), and 41 (C_3_H_5_^+^) which were consistent with those of monounsaturated C_14_ aliphatic acetates. The mass spectra of peaks A, B, C, and D respectively matched those of *Z*9-14:OH, *Z*5-14:Ac, *Z*7-14:Ac, and *Z*9-14:Ac in the NIST11 library. Finally, in comparative GC-MS analyses with authentic standards the identity of the four compounds was further confirmed by the matching of retention times and fragmentation patterns. In the hexane extracts from one female abdominal tip, on the average, 27.20 ± 5.47 ng of *Z*9-14:Ac, 2.81 ± 0.15 ng of *Z*5-14:Ac, 1.76 ± 0.18 ng of *Z*7-14:Ac and 1.23 ± 0.06 ng of *Z*9-14:OH were found. In the SPME extracts from the head-space of individual abdominal tips, *Z*9:14:Ac, *Z*5-14:Ac, *Z*7-14:Ac, and *Z*9:14 OH were detected in the ratio of 82:9:5:4.

**Table 1:** Mass spectral data of components (A, B, C, D) of sex pheromone gland extracts and external standard compounds (ES)

Component	Identity	m/z (relative intensity of major ions) [assignment]
A	*Z*9-14:OH	194 (3) [M^+^–H_2_O], 166 (2) [M^+^–C_2_H_5_OH], 109 (17), 96 (37), 95 (37), 82 (58), **81** (55), 69 (35), 68 (36), 67 (56), 55 (100), 54 (37), 41 (55), 31 (15) [CH_2_OH^+^]
B	*Z*5-14:Ac	194 (9) [M^+^–CH_3_COOH], 166 (7) [M^+^–CH_3_COOC_2_H_5_], 152 (1), 138 (8), 124 (8), 96 (35), 95 (40), 82 (58), **81** (45), 69 (24), 68 (41), 67 (63), 61 (4) [CH_3_COOH_2_^+^], 55 (40), 54 (39), 43 (100) [O = CCH_3_^+^], 41 (46)
C	*Z*7-14:Ac	194 (10) [M^+^–CH_3_COOH], 166 (2) [M^+^–CH_3_COOC_2_H_5_], 152 (1), 138 (5), 124 (7), 123 (8), 110 (15), 109 (21), 96 (44), 95 (39),83 (20), 82 (56), **81** (57),69 (32), 67 (65), 68 (35), 61 (7) [CH_3_COOH_2_^+^], 55 (54), 54 (37), 43 (100) [O = CCH_3_^+^], 41 (50)
D	*Z*9-14:Ac	194 (14) [M^+^–60], 152 (2), 110 (23), 96 (55), 95 (45), 82 (71), **81** (67),69 (33), 68 (35), 67 (64), 61 (10) [CH_3_COOH_2_^+^], 55 (81), 54 (38), 43 (100) [O = CCH_3_^+^], 41 (52)
ES	*Z*7-12:Ac	166 (21) [M^+^–CH_3_COOC_2_H_5_], 152 (2), 138 (7), 124 (9), 96 (66), 95 (55), 82 (86), **81** (97), 69 (36), 68 (51), 67 (100), 61 (9) [CH_3_COOH_2_^+^], 55 (78), 54 (51), 43 (78) [O = CCH_3_^+^], 41 (46)

m/z 81 quantifier ion

### EAG Responses

**Fig. 3:**
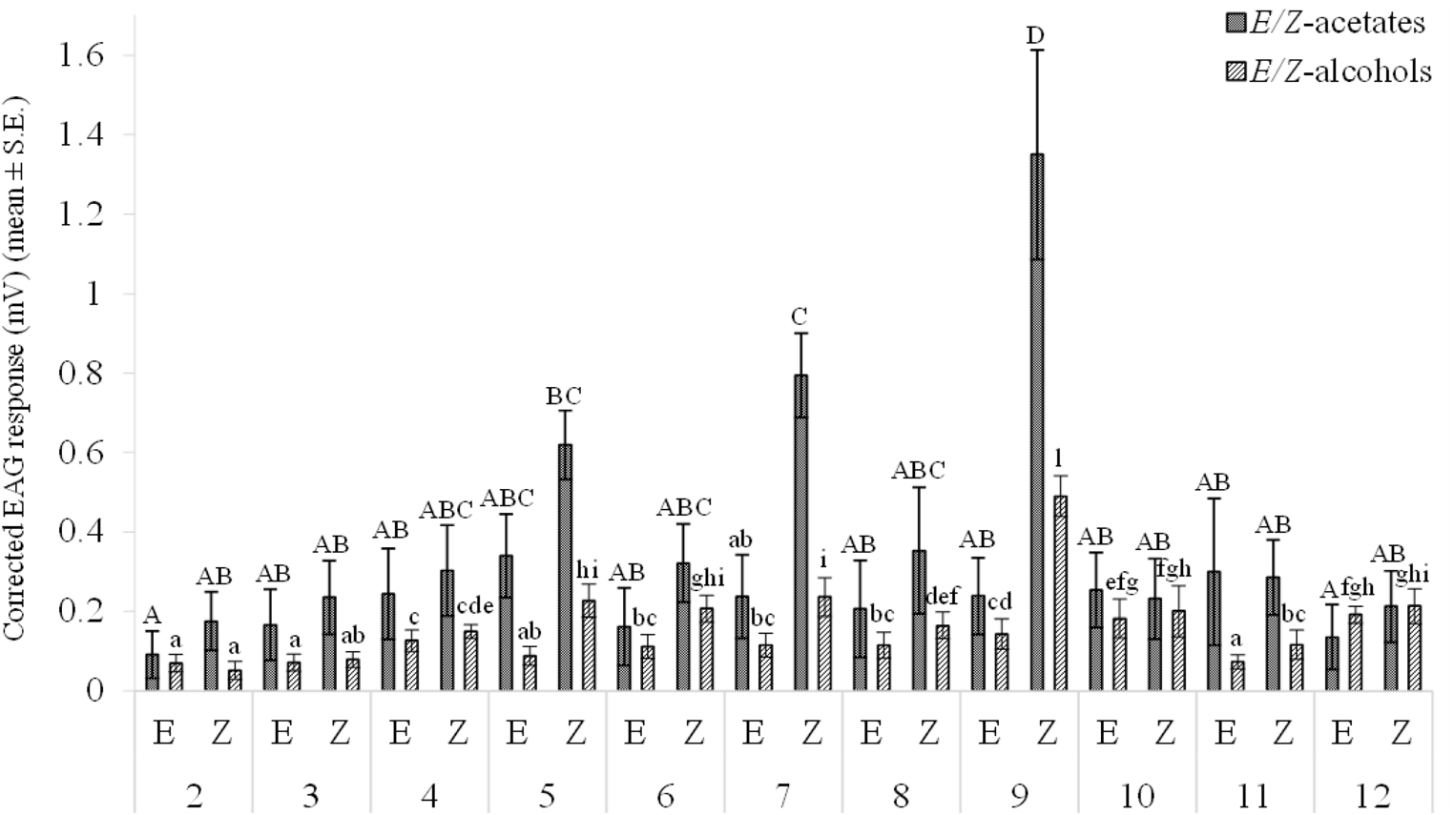
EAG responses of male *P. caestrum* antennae (*n* = 3) to (*Z*) and (*E*) isomers of monounsaturated C_14_ aliphatic acetates and alcohols. Bars with different letters are significantly different at *P* = 0.05 (Tukey test)

The EAG responses of male *P. caestrum* antennae to a series of *Z* and *E* isomers of monounsaturated C_14_ aliphatic acetates and alcohols are reported in (Fig. 3). Among the mean EAG responses to the *Z* and *E* isomers of monounsaturated C_14_ acetates significant differences (F = 7.63, df = 21, *P* < 0.001) were found with *Z*9-14:Ac, *Z*7-14:Ac, and *Z*5-14:Ac ranking, in decreasing order, as the most EAG-active compounds. Significant differences were found among the mean EAG responses to the *Z* and *E* isomers of monounsaturated C_14_ alcohols (F = 136.12, df = 21, *P* < 0.001) with *Z*9-14:OH being the strongest antennal stimulant (Fig. 3).

**Table 2:** Mean trap catches of male *P. caestrum* in funnel traps (Novatrap, Novapher, Italy) baited with candidate sex pheromone components singly and in various combinations loaded (1000 µg) on green rubber septum dispensers

Components of bait (µg)				Mean (± S.E.) trap catches^a^
Z9-14:Ac	Z9-14:OH	Z5-14:Ac	Z7-14:Ac	
1000				0.0 a
	1000			0.0 a
		1000		0.0 a
			1000	0.0 a
850	150	0	0	0.0 a
850	0	0	150	3.33 ± 0.88 a
850	0	150	0	6.33 ± 1.20 b
850	0	100	50	86.67 ± 10.65 c
850	0	50	100	6.67 ± 1.05 b
850	50	100	0	0.67 ± 0.08 b
850	50	0	100	0.33 ± 0.02 a
800	50	100	50	5.67 ± 0.87 b
800	50	50	100	0.0 a
Blank trap				0.0 a

^a^ Mean trap catches followed different letters are significantly different at *P* = 0.05 (Tukey test)

**Fig. 4:**
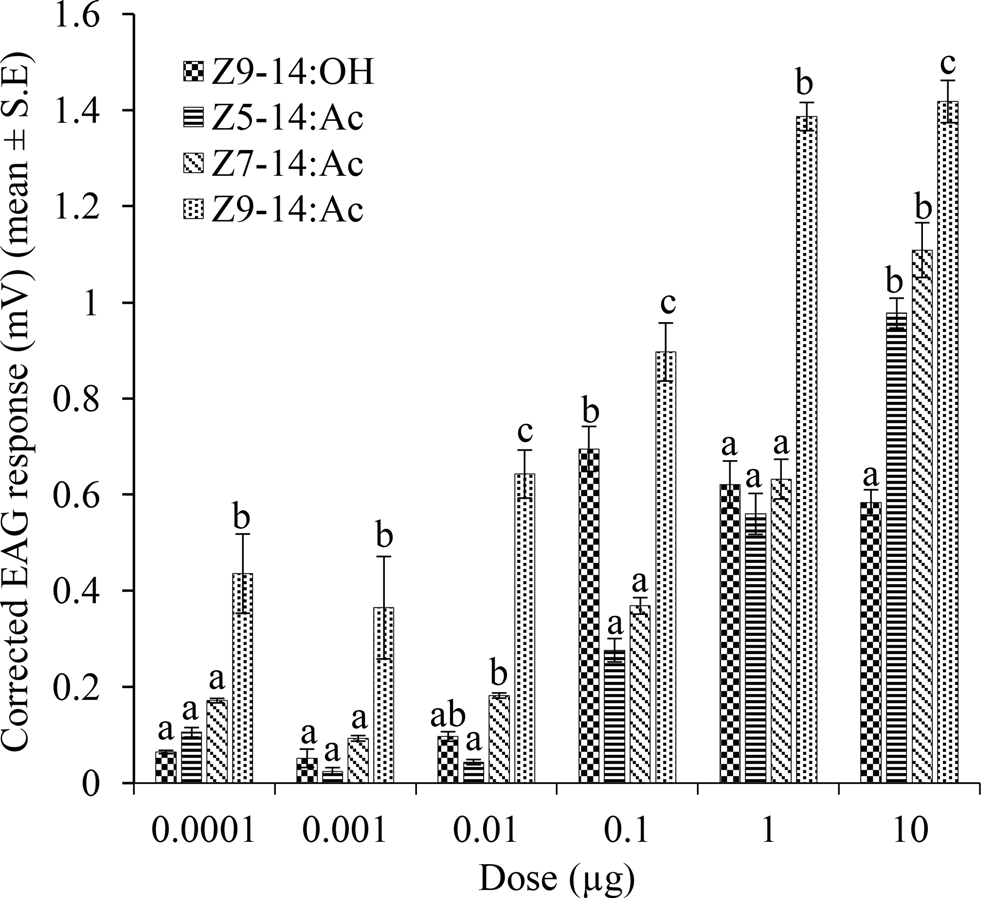
Mean EAG responses (± SE) of *P. caestrum* males (*N* = 5) to the EAD-active compounds identified in the sex pheromone gland of calling females. In the same dose group, bars with the same letter are not significantly different at *P* = 0.05 (Tukey test)

The male EAG responses to different doses of the four putative sex-pheromone components are reported in Fig. 4. In the dose range tested, the mean EAG response varied from 0.43 to 1.42 mV for *Z*9-14:Ac, from 0.17 to 1.11 mV for *Z*7-14:Ac, from 0.11 to 0.98 mV for *Z*5-14:Ac and from 0.11 to 0.80 mV for *Z*9-14:OH. At different dose tested, the mean EAG response to *Z*9-14:Ac was significantly (F = 8.26–142.39; df = 3; *P* = 0.002 – <0.001) higher than those to the other compounds (*P* < 0.05, Tukey-test). Finally, the amplitude of the mean EAG response to *Z*9-14:OH decreased from the 0.1 to 10 µg dose, indicating saturation of the antennal receptors at the 0.1 µg dose.

### Field Tests

**Fig. 5:**
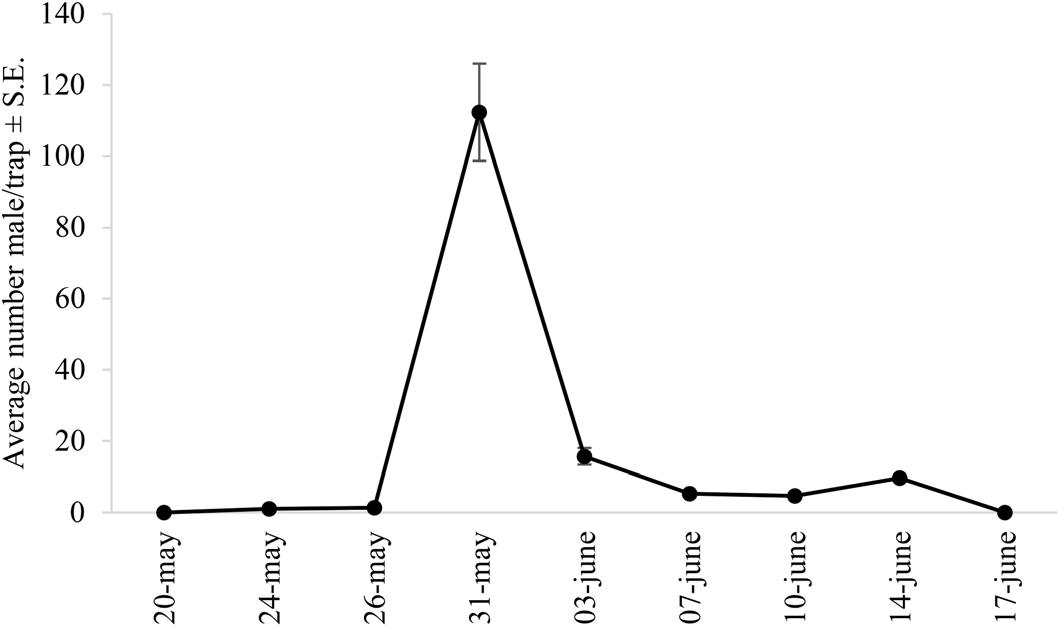
Flight activity of *P. caestrum* males as monitored by funnel traps (Novatrap, Novapher, Italy) baited with 1000 µg of a 85:5:10 blend of *Z*9-14:Ac, *Z*7-14:Ac, and *Z*5-14:Ac in Foggia (Italy) from 18 May to 20 June 2022

During field tests, 429 males of *P. caestrum* were caught from 20 May to 20 June. The three-component blend containing *Z*9-14:Ac, *Z*7-14:Ac, and *Z*5-14:Ac in a 85:5:10 ratio attracted a significantly (F = 10.40; df = 13; *P* < 0.001) higher number of males than the other lures (Table 1). The 85:15 ratio of *Z*9-14:Ac and *Z*5-14:Ac, the 85:10:5 ratio of *Z*9-14:Ac, *Z*7-14:Ac, and *Z*5-14:Ac and the 80:5:10:5 ratio of *Z*9-14:Ac, *Z*7-14:Ac, *Z*5-14:Ac, and *Z*9-14:OH were significantly less attractive than the most effective ternary blend but allowed for capturing significantly more males than the remaining lures. Male captures by single components and the remaining binary, ternary, and quaternary blends were not significantly different and similar to those obtained by unbaited traps (Table 1). Traps baited with the effective three-component blend did not catch males of other moth species. Monitoring the emergence of *P. caestrum* males by this lure showed one period of flight activity occurring from the third decade of May to the second decade of June with a peak at the end of May (Fig. 5).

## Discussion

*Parahypopta caestrum* virgin females exhibited the typical calling posture, evidenced by the extrusion of last urites, during the first hour of the first scotophase thus indicating that females are sexually mature shortly after emergence.

GC-MS-EAD analysis of hexane and SPME extracts from female abdominal tips during the first hour of calling detected the presence of four compounds eliciting antennal responses in *P. caestrum* males. According to their retention times, mass spectra, and comparative EAG analyses with synthetic standards, these active compounds were identified as *Z*9-14:Ac, *Z*7-14:Ac, *Z*5-14:Ac, and *Z*9-14:OH.

Field trapping tests showed that all the three monounsatured C_14_ aliphatic acetates are essential for attracting male moths. *Z*9-14:Ac was the most abundant in hexane and SPME gland extracts and triggered the strongest EAG responses in male moths but was not attractive by itself and when tested in combination with *Z*7-14:Ac or *Z*9-14:OH in a 85:15 ratio. Among binary blends, a slight male attraction was elicited by the 85:15 ratio of *Z*9-14:Ac and *Z*5-14:Ac. Among the ternary blends tested, the 85:5:10 ratio of *Z*9-14:Ac, *Z*7-14:Ac, and *Z*5-14:Ac was shown to be optimal for the attraction of *P. caestrum* males. In fact, reducing the proportion of *Z*5-14:Ac and increasing that of *Z*7-14:Ac from the 85:5:10 blend or substituting one of them with *Z*9-14:OH resulted in a reduction of male attraction. These results strongly suggested that all three compounds, in this precise ratio, act as a unit in eliciting the male response both at long and short distances (Linn et al. 1987; Sans et al. 1997; Germinara et al. 2007).

The identified sex pheromone components of *P. caestrum* have been reported also as active sex pheromone components in many species of Lepidoptera (El Sayed 2023). In Cossidae, *Z*9-14:Ac is also the main sex-pheromone component of *C. tristis* (Bouwer et al. 2015) and *I. sibirica*, another destructive pest of *A. officinalis*, widely distributed in the Siberia, Mongolia and north and north-east of China (Zhang et al. 2011). In this latter, *Z*9-14:Ac is in combination with *Z*7-14:Ac, both compounds are necessary for attraction and the addition of (*Z*)-9-hexadecenyl acetate to a 10:5 blend of the two components resulted in a significant increase of male catches (Zhang et al. 2011). *Z*7-14:Ac has been identified as the main sex-pheromone component of *H. vicarious* (Yang et al. 2015) and *H. arenicola* (Jing et al. 2010) and as one of the two active components of the *H. hippophaecolus* sex pheromone (Fang et al. 2005). As regard *Z*5-14:Ac, which is a secondary active component of *C. cossus* (Capizzi et al. 1983) and *H. arenicola* sex pheromones (Jing et al. 2010), it elicited high EAG responses in *I. sibirica* males but did not enhance male trap catches when added to the blend of active components (Zhang 2011).

The antagonistic role of *Z*9-14:OH in attracting male *P. caestrum* moths remain to be further elucidated. Biosynthetically *Z*9-14:OH could be a precursor of the corresponding acetate in sex pheromone glands (Tillman et al. 1999). The presence in the abdominal tips of virgin female moths of alcohol compounds inhibiting male attractions to sex pheromone has also been observed for other species including *Lymantria dispar* (L.) (Cardé et al. 1973), *Coristoneura fumiferana* (Clemens) (Weatherston and Maclean 1974), and *Helicoverpa armigera* (Hübner) (Xu et al. 2016). In this latter, it was postulated that the pheromone antagonist *Z*11-16:OH acts as a regulatory signal to indicate optimal mating times, and that its detection by males prevent mating with immature females (Chang et al. 2017). In this study, GC-MS-EAD analysis and EAG recordings showed that *Z*9-14:OH is detected by male *P. caestrum* antennae however actual emission of this compounds by living virgin females along with possible variations in the blend ratio between the alcohol and corresponding acetate during different scotophases require to be investigated to demonstrate that females use *Z*9-14:OH to signal their sexual maturity.

Trapping of *P. caestrum* males by the ternary blend of *Z*9-14:Ac, *Z*7-14:Ac, and *Z*5-14:Ac in the 85:5:10 blend allowed for a timely monitoring of adult emergence and provided accurate information on their flight activity. These data indicated that in the area of study (southern Italy), adult moths are present in asparagus fields for a short period of approximately three weeks from the end of May to the middle of June.

In conclusion, green rubber septum dispensers baited with 1 mg of a 85:5:10 ratio of *Z*9-14:Ac, *Z*7-14:Ac, and *Z*5-14:Ac provides an effective tool for detecting and monitoring *P. caestrum*. Field evidence on the attractiveness of this blend encourage extensive field trials to determine optimum pheromone dosage and trap for the development of mass trapping as a non-insecticidal control strategy against this pest.

## Data Availability

No datasets were generated or analysed during the current study.
